# The benefit of immunonutrition in patients undergoing hepatectomy: a systematic review and meta-analysis

**DOI:** 10.18632/oncotarget.20045

**Published:** 2017-08-08

**Authors:** Chengshuo Zhang, Baomin Chen, Ao Jiao, Feng Li, Bowen Wang, Ning Sun, Jialin Zhang

**Affiliations:** ^1^ Hepatobiliary Surgery Department and Unit of Organ Transplantation, First Hospital of China Medical University, Shenyang 110001, Liaoning, P.R. China

**Keywords:** hepatectomy, immunonutrition, ω-3 fatty acids, randomized controlled trials, meta-analysis

## Abstract

Perioperative immunonutrition in liver resection remains doubtful. A systematic review and meta-analysis was conducted to compare postoperative outcomes between patients undergoing hepatectomy who received perioperative immunonutrition and those who did not.A PubMed, Embase, Cochrane Central Register of Controlled Trials, and Web of Knowledge database search was performed to retrieve all of the randomized controlled trials (RCTs) evaluating the value of perioperative immunonutrition in patients undergoing hepatectomy until the end of September 2016. Data extraction and quality assessment of RCTs were performed in accordance with PRISMA guidelines. The quality of evidence for each postoperative outcome was assessed using the GRADEpro analysis. A random-effects model was used to conduct a meta-analysis with RevMan 5.3.5 software. Eight RCTs including 805 patients (402 with and 403 without immunonutrition) were identified. Immunonutrition, mainly ω-3 fatty acids, significantly reduced the incidence of postoperative total complications (risk ratio [RR] = 0.59; 95% confidence interval [CI], 0.46–0.75; *p* < 0.0001) and infectious complications (RR = 0.46; 95% CI, 0.32–0.68; *p* < 0.0001), and shortened the length of hospital stay (standardized mean difference, −0.49; 95% CI, −0.81 to −0.16; *p* = 0.0004). There was no significant between-group difference in postoperative mortality (RR = 0.46; 95% CI, 0.16–1.31; *p* = 0.15). Immunonutrition, mainly ω-3 fatty acids, is potentially beneficial in reducing overall and infectious postoperative complications and in shortening the hospital stay for patients undergoing hepatectomy.

## INTRODUCTION

Hepatectomy is the principal treatment for eligible patients with benign and malignant hepatobiliary diseases. Although improvements in operative techniques and perioperative management have markedly ameliorated the prognosis of patients undergoing hepatectomy [1], the rate of postoperative complications remains high, as shown by longer hospital stay and increased mortality [2]. Strategies are therefore needed to reduce the incidence of complications following hepatic resection.

Immunonutrition refers to enteral and parenteral nutritional formulas that mainly include ω-3 polyunsaturated fatty acids (FAs), arginine, glutamine, and nucleotides [3]. Patients are administered immunonutrition to increase their nitrogen balance and protein synthesis, modulate postsurgical immunosuppression and inflammatory responses, and improve the host immune state [4, 5]. Immunonutrition support in patients undergoing major elective upper gastrointestinal (GI) surgery has been found to improve their immunologic, metabolic, and clinical outcomes [6]. The results of more than 28 randomized trials and many available meta-analyses have led many academic societies to recommend immunonutrition [7]. For example, the guidelines of the American Society for Parenteral and Enteral Nutrition (ASPEN) and the European Society for Clinical Nutrition and Metabolism (ESPEN) have recommended immunonutrition in patients undergoing abdominal operations, especially those with upper GI cancer [8, 9].

Immunonutrition has been introduced perioperatively and extensively to subjects undergoing hepatic resection, including patients with primary and secondary liver neoplasms and living liver transplant donors. Several randomized controlled trials (RCTs) have shown that immunonutrition controls inflammatory responses, protects liver function, and promotes liver regeneration, while markedly reducing postoperative complications, length of stay (LoS) in hospital, and mortality [10–16]. Other studies, however, have yielded conflicting results [17–19]. To our knowledge, no systematic review and meta-analysis has assessed the effects of perioperative immunonutrition support in patients undergoing hepatectomy. This systematic review and meta-analysis therefore compared postoperative outcomes in patients enrolled in RCTs who underwent hepatectomy with and without immunonutrition.

## RESULTS

### Study identification and selection

**Figure 1:**
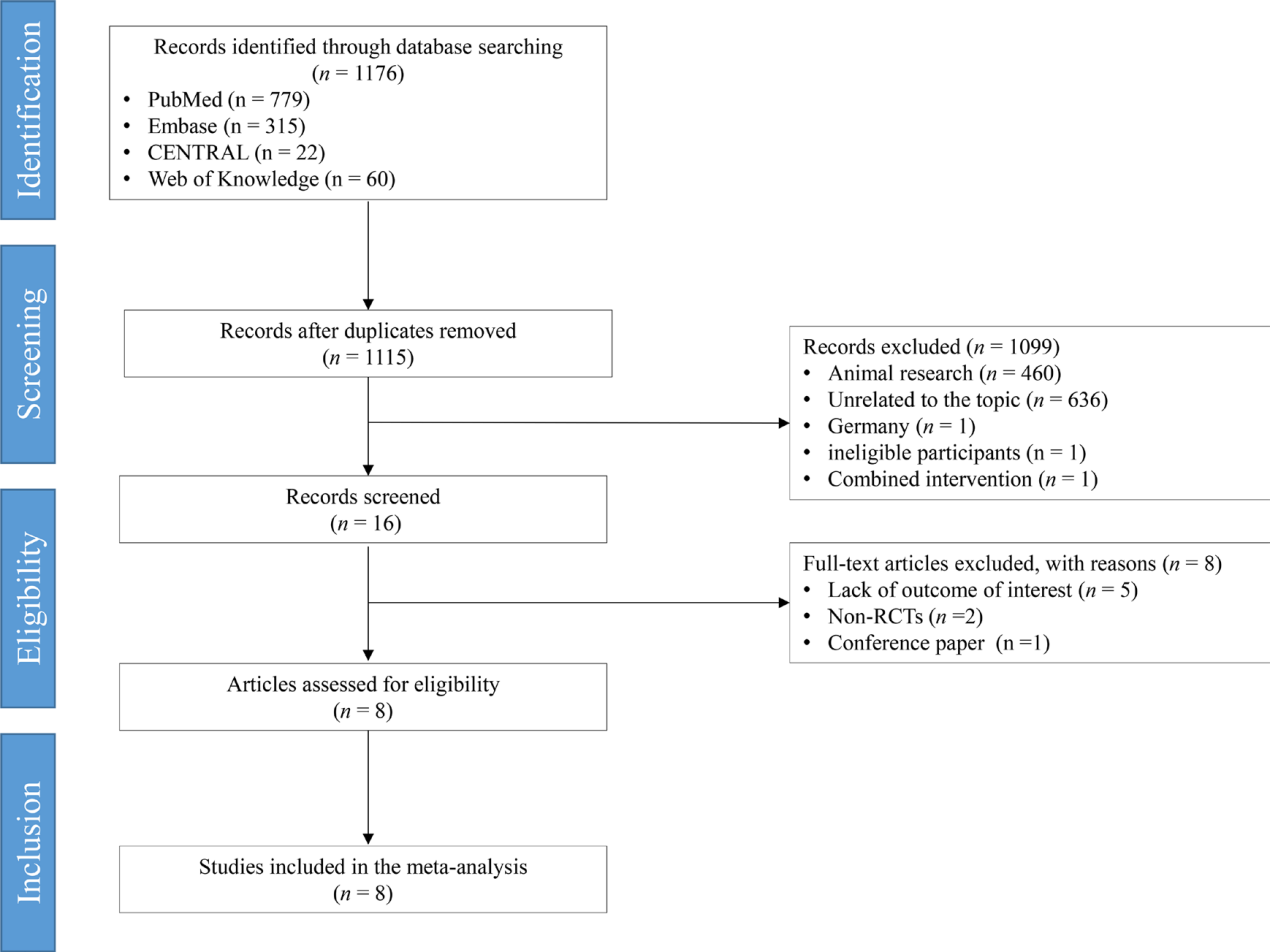
Preferred reporting items for systematic reviews and meta-analyses flowchart RCT, randomized controlled trial.

Based on the search terms, we primarily retrieved a total of 1176 potential articles. Sixty-one duplicates were excluded using EndNote X7.7 (Thomson Reuters, MI). A total of 1099 citations were excluded after screening their titles and abstracts. After screening the full text of 16 remaining articles, 8 ineligible trials were excluded because of several reasons such as being non-RCTs, conference papers, and lacking outcomes of interest. All procedures were performed independently by 2 investigators. Finally 8 articles [12–19] were selected and incorporated into this meta-analysis (Figure 1).

### Study characteristics

All eligible studies were published between 2011 and 2016 and included a total of 805 patients (598 men and 207 women). Of these patients, 402 received immunonutrition supplementation and 403 did not. The average number of patients per study was 101 (range 35–320). Four studies [13, 17–19] performed per-protocol analysis and one [14] conducted intention-to-treat analysis. Standard criteria for inclusion and exclusion were defined in all included studies. Two studies [13, 19] reported 30-day evaluations after surgery and three [14, 15, 17] described 6-month follow-up.

Timing, duration, type, and dosage of immunonutrition varied between studies. Immunonutrition was given preoperatively for a minimum of 5 days in 2 studies [14, 18], postoperatively for 5–7 days in 5 studies [12, 13, 15–17], and perioperatively in 1 study [19]. Postoperative supplementation altered from the day after surgery to the day of hemodynamic stability. Perioperative supplementation was commenced 7 days before surgery and was continued postoperatively for 3 days. Immunonutrition used in these studies comprised mainly ω-3 FAs and other immune-modulating nutrients such as arginine and nucleotides. In 6 trials [12–17], immunonutrition was described as ω-3 FAs administered via the parenteral or enteral route while 2 studies [18, 19] specified the oral immunonutrition diet IMPACT containing ω-3 FAs, arginine, and nucleic acids. An isocaloric diet was used as a control diet in all studies and an isonitrogenous diet was set as a control diet in 3 studies [15–17]. Enteral feeding was administered by peroral route and nasogastric tube in 3 studies [14, 18, 19]. The target dosage for preoperative supplementation was 900 mL/d and 750 mL/d, respectively, in 2 studies [18, 19]. Target calories for postoperative supplementation were calculated as kcal/kg/d (25–35 kcal/kg) in 5 studies [12, 13, 15–17]. The main characteristics of the 8 included RCT studies are summarized in Supplementary Table 1.

### Assessment of bias risk

**Figure 2:**
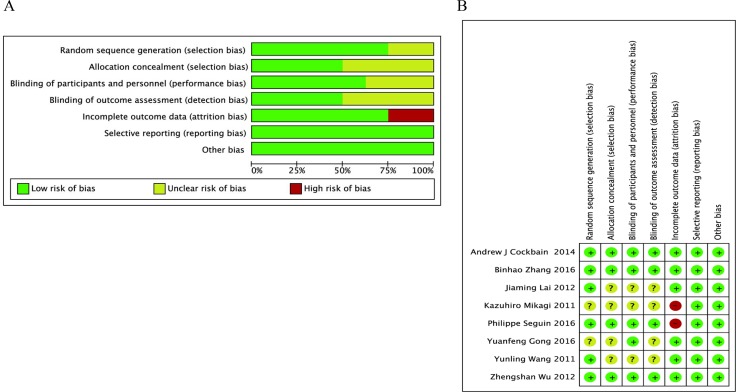
Assessment of risk of bias (**A**) Risk of bias graph. (**B**) Risk of bias summary.

The bias risk of the included RCTs was critically analyzed using the Cochrane Collaboration risk of bias tool. Six studies [13–17, 19] described the methods of patient randomization, 4 [13–15, 19] properly reported concealment of allocation, 5 [12–15, 19] described blinding of participants and personnel, and 4 [13–15, 19] mentioned an assessor of outcomes. Of the 5 studies [13, 14, 17–19] that reported dropouts after randomization, 2 [18, 19] were regarded as high risk because the dropout rate was > 20%. Selective outcome reporting and other sources of bias were not identified. The risk of bias in each study is shown in Figure 2.

### Effect of immunonutrition on postoperative total complications

**Figure 3:**
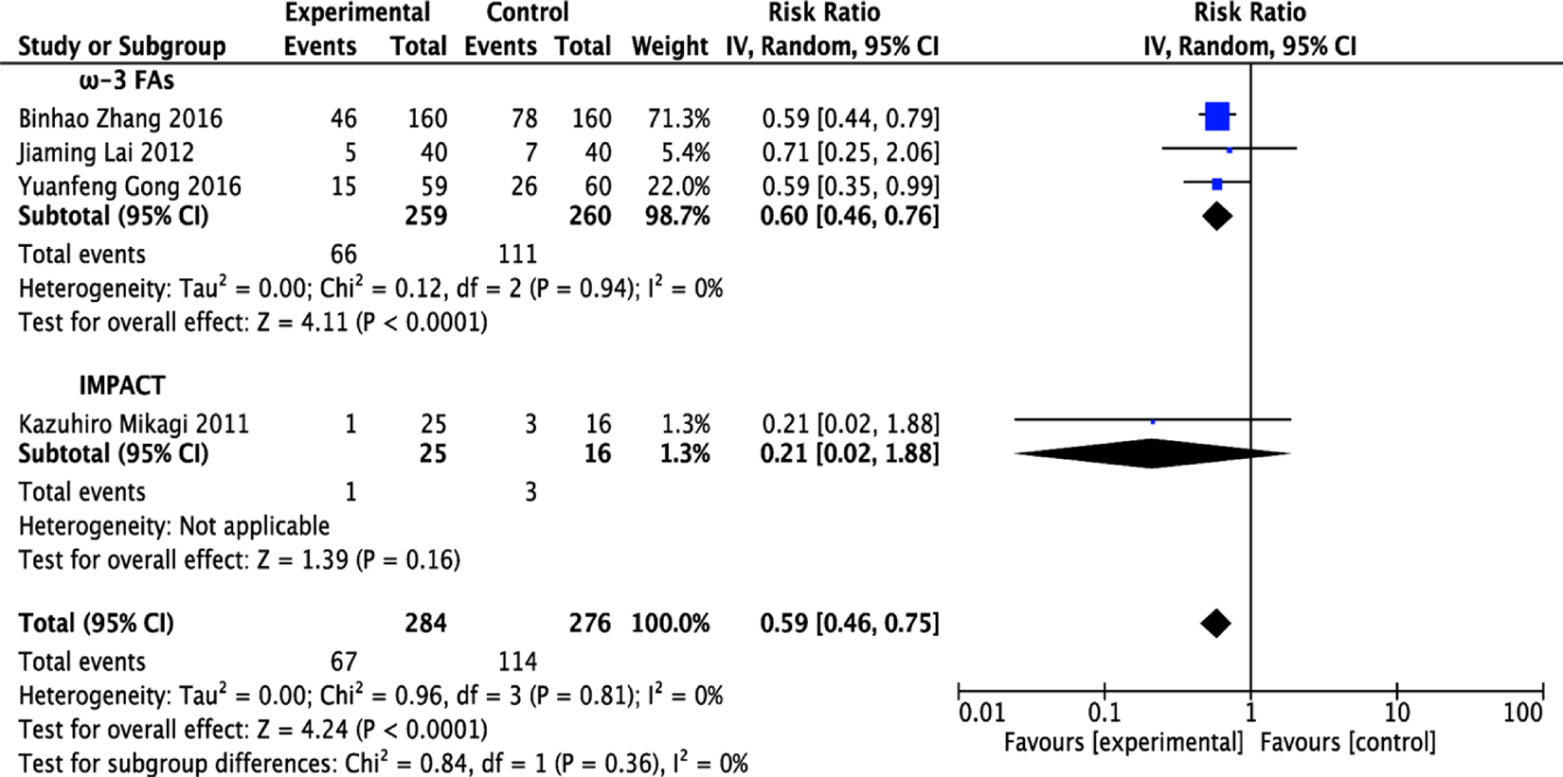
Forest plot of pooled data on postoperative total complications CI, confidence interval; df, degrees of freedom; IV, inverse variance (statistical method).

Four RCTs [12, 13, 17, 18] evaluated the rates of postoperative total complications; these trials included 284 patients who received immunonutrition and 276 patients who did not. Pooled analysis showed that immunonutrition significantly reduced the incidence of postoperative total complications (risk rate (RR) = 0.59; 95% confidence interval (CI), 0.46–0.75; *p* < 0.0001). The chi-squared test for heterogeneity was not significant (*p* = 0.81, *I*^2^ = 0 %) (Figure 3 and Table 1).

**Table 1:** Subgroup analysis based on component of perioperative immunonutrition support in patients undergoing hepatectomy^1^

Outcomes	Studies [ref]	No. of patients	Statistical method	Effect size (95% CI)	Test for overall effect (*P* value)^2^
**Postoperative total complications**					
ω-3 FAs	3 [12, 13, 17]	519	IV (Random) RR	0.60 [0.46–0.76]	*p* < 0.0001
IMPACT	1 [18]	41	IV (Random) RR	0.21 [0.02–1.88]	*p* = 0.16
Immunonutrition	4 [12, 13, 17, 18]	560	IV (Random) RR	0.59 [0.46–0.75]	*p* < 0.0001
**Postoperative infectious complications**					
ω-3 FAs	4 [12, 13, 15, 17]	582	IV (Random) RR	0.49 [0.33–0.73]	*p* = 0.0004
IMPACT	2 [18, 19]	76	IV (Random) RR	0.26 [0.07–0.95]	*p* = 0.04
Immunonutrition	6 [12, 13, 15, 17–19]	658	IV (Random) RR	0.46 [0.32–0.68]	*p* < 0.0001
**Length of hospital stay**					
ω-3 FAs	5 [12, 13, 15–17]	664	IV (Random) SMD	−0.49 [−0.81 to −0.16]	*p* = 0.004
**Postoperative mortality**					
ω-3 FAs	5 [12–15, 17]	670	IV (Random) RR	0.46 [0.16–1.31]	*p* = 0.15

^1^ CI, confidence intervals; IV, inverse variance; ref, reference; RR, risk ratio; SMD, standardized mean difference.

^2^ *P* value: based on test for overall effect in the meta-analysis (*Z* test).

Stratified meta-analysis was performed to show the effect of ω-3 FA-enriched and IMPACT formulas. Results demonstrated that ω-3 FA-containing formulas significantly attenuated postoperative total complications in patients undergoing hepatectomy (RR = 0.60; 95% CI, 0.46–0.76; *p* < 0.0001). The chi-squared test for heterogeneity was not significant in this subgroup (*p* = 0.94, *I*^2^ = 0 %). Regarding IMPACT formulas, the risk was reduced but the result was not significant (RR = 0.21; 95% CI, 0.02–1.88; *p* = 0.16), and it was not necessary to check heterogeneity in this subgroup comprising 1 study (Figure 3 and Table 1).

### Effect of immunonutrition on postoperative infectious complications

**Figure 4:**
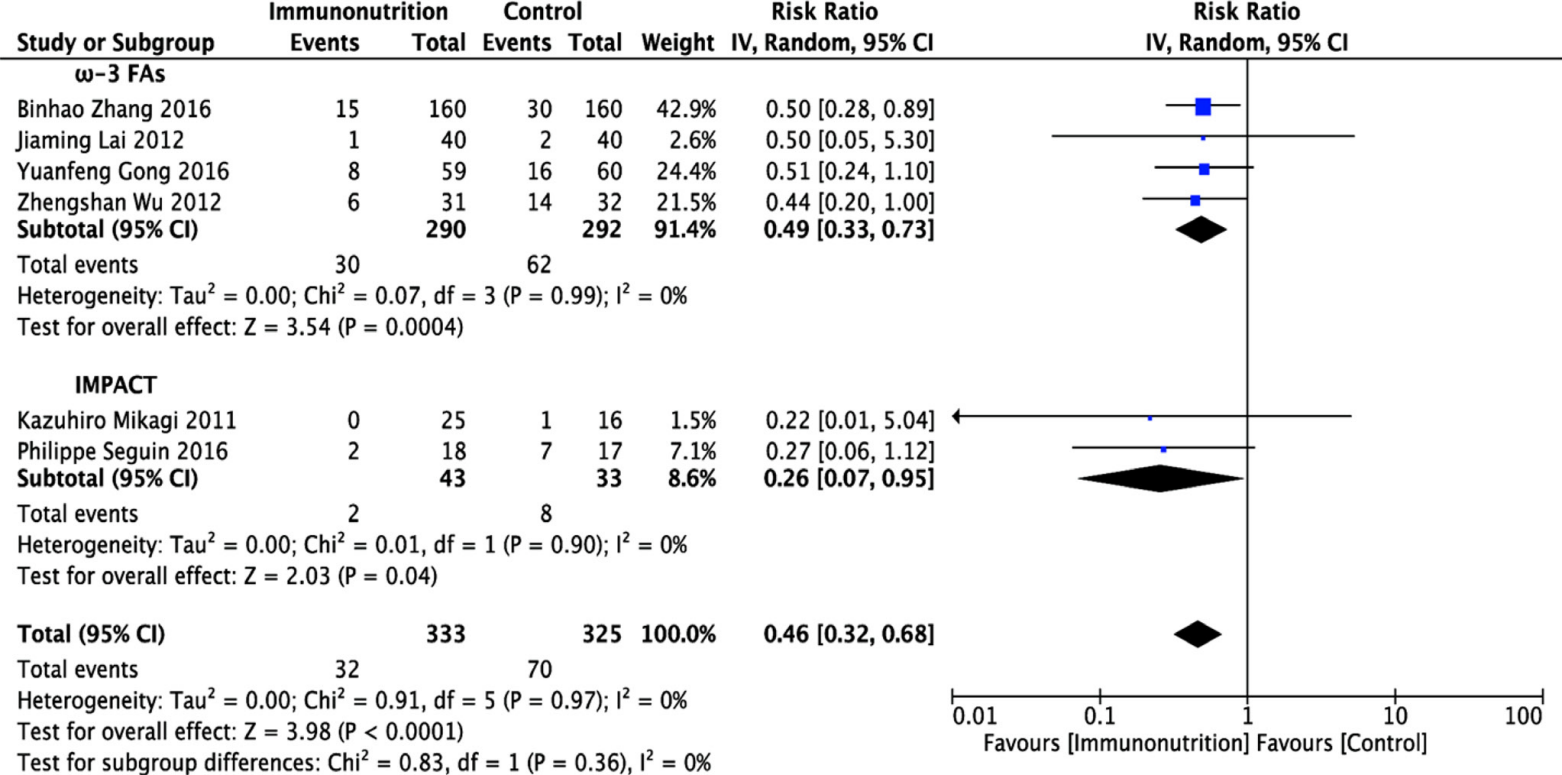
Forest plot of pooled data on postoperative infectious complications CI, confidence interval; df, degrees of freedom; IV, inverse variance (statistical method).

Six [12, 13, 15, 17–19] of the 8 studies reported postoperative infectious complications; these studies included 333 patients in the immunonutrition group and 325 patients in the control group. Pooled analysis showed that immunonutrition significantly reduced the incidence of infectious complications in patients undergoing hepatectomy (RR = 0.46; 95% CI, 0.32–0.68; *p* < 0.0001). The chi-squared test for heterogeneity was not significant (*p* = 0.97, *I*^2^ = 0%) (Figure 4 and Table 1).

Stratified meta-analysis was conducted to show the effect of ω-3 FA-enriched and IMPACT formulas separately. Results demonstrated that both ω-3 FA-enriched and IMPACT formulas significantly reduced the incidence of infectious complications in patients undergoing hepatectomy (ω-3 FAs: RR = 0.49; 95% CI, 0.33–0.73; *p* = 0.0004; IMPACT: RR = 0.26; 95% CI, 0.07–0.95; *p* = 0.04). No evidence of between-study heterogeneity was found in ω-3 FA (*p* = 0.99, *I*^2^ = 0%) and IMPACT subgroups (*p* = 0.90, *I*^2^ = 0%) (Figure 4 and Table 1).

### Effect of ω-3 FA-enriched formulas on length of hospital stay

**Figure 5:**
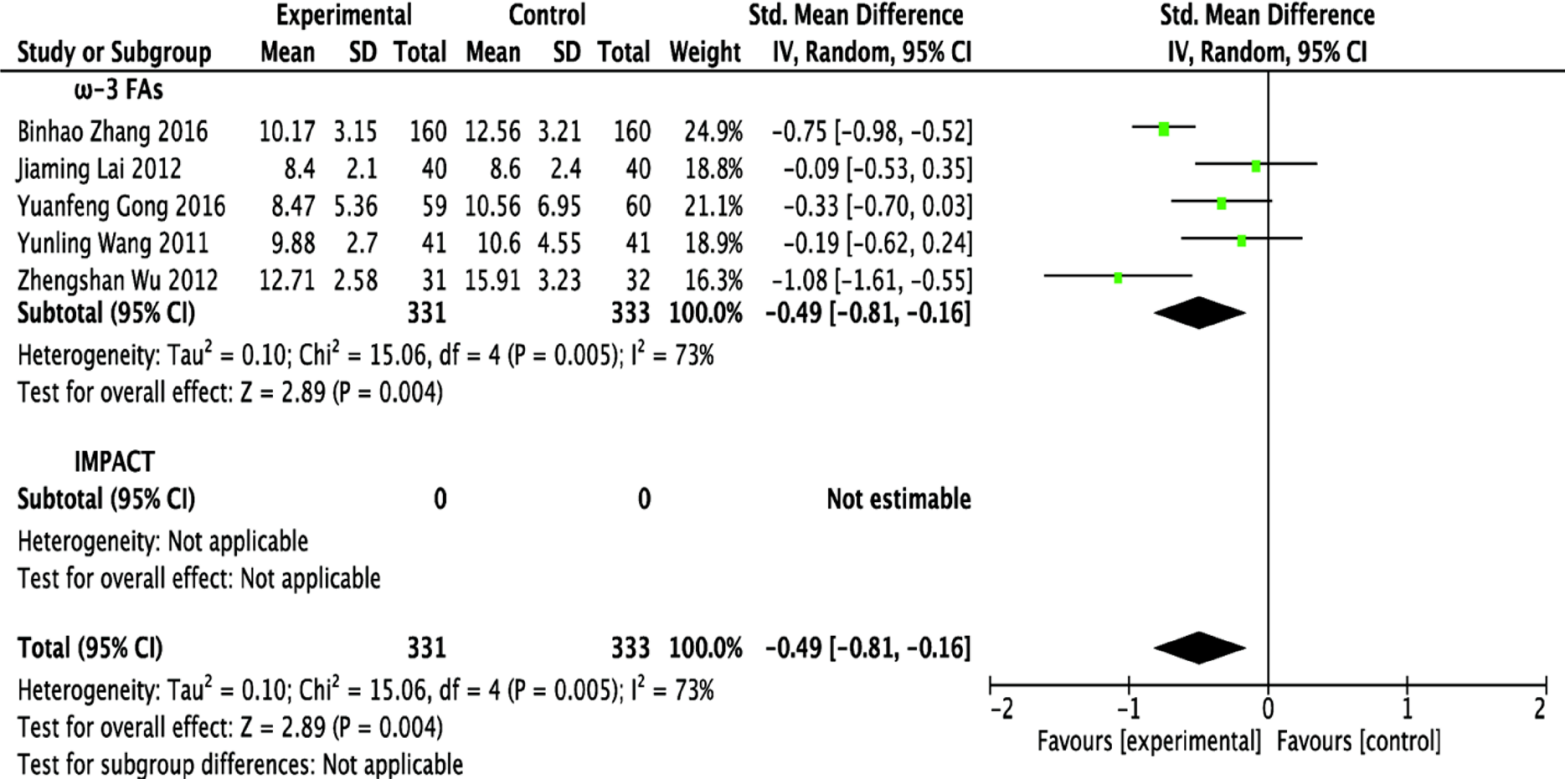
Forest plot of pooled data on length of hospital stay CI, confidence interval; df, degrees of freedom; IV, inverse variance (statistical method).

Five RCT studies [12, 13, 15–17] reported hospital LoS as mean ± standard deviation (SD), with corresponding effect size expressed as standardized mean difference (SMD) with 95% CI. Mean difference was set as SMD because of the record of perioperative hospital stay in 2 studies [13, 15] and postoperative hospital stay in 3 studies [12, 16, 17]. In 2 other studies [14, 18], LoS was expressed as median with range or mean only. Five studies included 331 patients receiving ω-3 FA-enriched formulas and 333 patients receiving the control formula. Mean LoS ranged from 8.4 to 12.71 days in the former group and from 8.6 to 15.91 days in the latter. A meta-analysis of included studies found that LoS was significantly shorter in the ω-3 FA-enriched group (SMD = −0.49; 95% CI, −0.81 to −0.16; *p* = 0.004), with significant statistical heterogeneity among studies (*p* = 0.005, *I*^2^ = 73%) (Figure 5 and Table 1).

### Effect of ω-3 FA-enriched formulas on postoperative mortality

**Figure 6:**
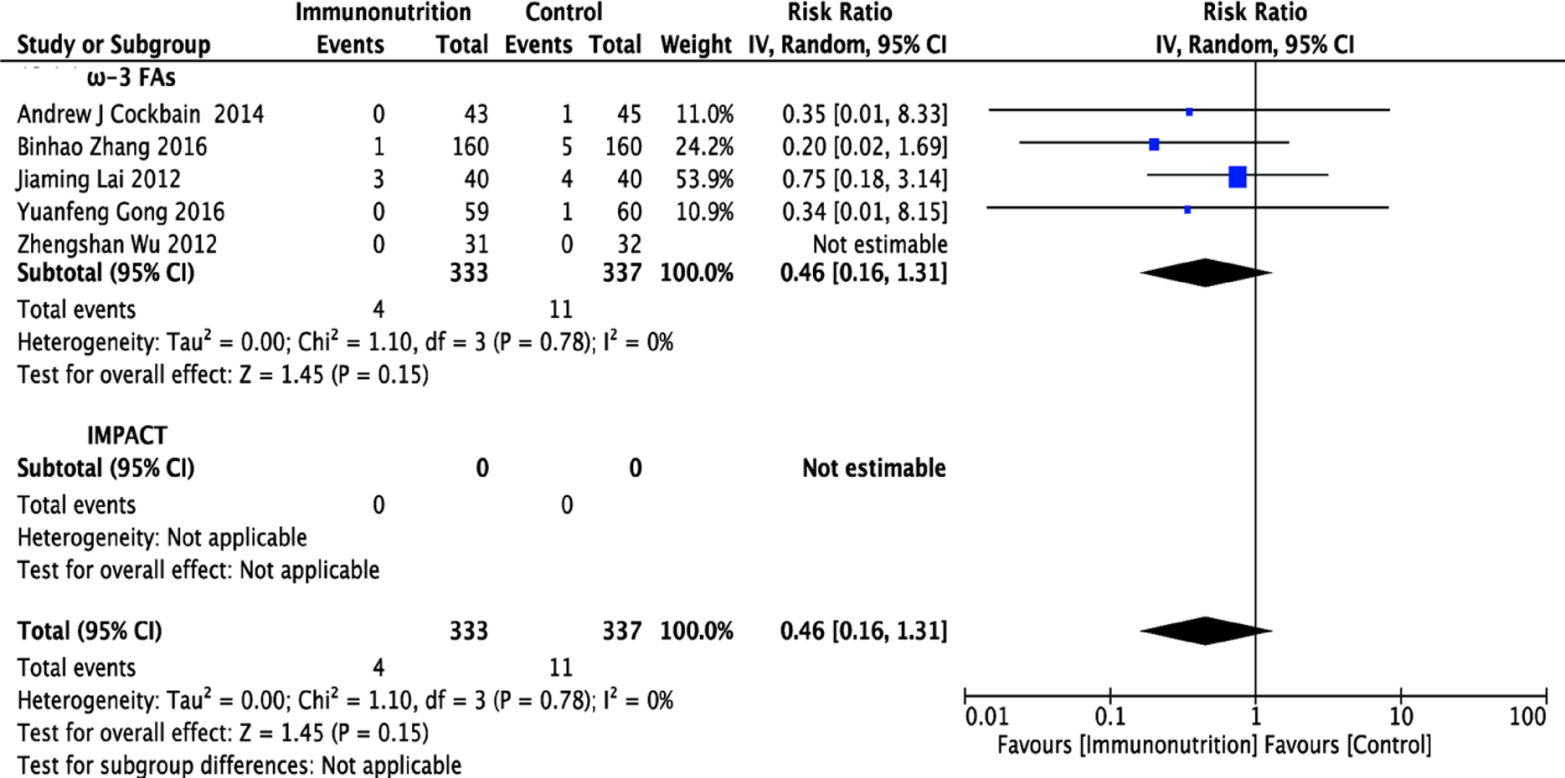
Forest plot of pooled data on postoperative mortality CI, confidence interval; df, degrees of freedom; IV, inverse variance (statistical method).

Five studies [12–15, 17], including 333 patients in the ω-3 FA-enriched group and 337 patients in the control group, reported postoperative mortality. Pooled analysis showed that mortality in the ω-3 FA-enriched group was lower than in the control group, but not significantly so (RR = 0.46; 95% CI, 0.16–1.31; *p* = 0.15). The chi-squared test for heterogeneity was not significant (*p* = 0.78, *I*^2^ = 0 %) (Figure 6 and Table 1).

### Analysis of the timing of immunonutrition

Subgroup analyses were also performed on the basis of the timing of initiation of immunonutrition. Postoperative immunonutrition was associated with a significant reduction in postoperative total complications, infectious complications, and hospital LoS. The results are displayed in Table 2.

**Table 2:** Analysis based on timing of initiation of the immunonutrition support in patients undergoing hepatectomy^1^

Outcomes	Preoperative immunonutrition	Perioperative immunonutrition	Postoperative immunonutrition
Studies [ref]	2 [14, 18]	1 [19]	5 [12, 13, 15–17]
Postoperative total complications(IV, random, RR, 95% CI)	0.21 [0.02–1.88] *P* = 0.16^2^	-	0.60 [0.46–0.74] *P* < 0.0001
Postoperative infectious complications(IV, random, RR, 95% CI)	0.22 [0.01–5.04] *P* = 0.34	0.27 [0.06–1.12] *P* = 0.07	0.49 [0.33–0.73] *P* = 0.0004
Length of hospital stay (IV, random SMD, 95% CI,)	-	-	−0.49 [−0.81 to −0.16] *P* = 0.004
Postoperative mortality (IV, random, RR, 95% CI)	0.35 [0.01–8.33] *P* = 0.52	-	0.47 [0.16–1.45] *P* = 0.19

^1^ CI, confidence intervals; IV, inverse variance; ref, reference; RR, risk ratio; SMD, standardized mean difference.

^2^ *P* value: based on test for overall effect in the meta-analysis (*Z* test).

### Quality assessment of evidence

The quality of evidence, as evaluated with GRADEpro, was ranked between moderate and very low as it was downgraded because of risk of bias, imprecision, and other considerations. The quality assessment of outcomes on the component of perioperative immunonutrition support in patients undergoing hepatectomy is shown in Supplementary Table 2.

## DISCUSSION

Hepatectomy is associated with high rates of postoperative complications and mortality, mainly due to the physiological, immunological, and metabolic responses to the pathological processes resulting from surgical intervention in the liver [20]. Theoretically immunonutrition should be beneficial, as certain nutritional components can regulate various inflammatory, metabolic, and immune processes. Omega-3 FAs, one of the pivotal components in immunonutrition formulas, has been found to modulate inflammation and immune reactions in tissue [21]. Arginine, another component of immunonutrition, not only upregulates immune function but improves nitrogen balance in patients under catabolic conditions [22]. A third component of immunonutrition, glutamine, is a plentiful free amino acid in the body and plays a vital role in integrating intestinal barrier function [23]. RNA can improve hypoimmunity and boost host defenses in cancer patients [24]. Taken together, these nutrients can reduce infectious complications, shorten hospital stay, and ameliorate prognosis in certain patients, including those undergoing GI surgery [25] and liver transplantation [26], as well as patients with critical illnesses [27] and acute pancreatitis [5]. To date, however, no systematic review and meta-analysis has been performed to assess whether patients benefit from immunonutrition support while undergoing hepatectomy.

This systematic review and meta-analysis based on 8 RCTs assessed the effects of immunonutrition against an isocaloric standard diet on clinical outcomes in patients undergoing hepatectomy for the first time. Perioperative immunonutrition was associated with a significant reduction in postoperative total complications, infectious complications, and length of hospital stay. On subgroup analysis based on the component of perioperative immunonutrition, a significant reduction in the incidence of postoperative total complications, infectious complications, and length of hospital stay were demonstrated after ω-3 FA supplementation. Infectious complications were also decreased with the addition of IMPACT. It should be noted that the role of glutamine cannot be defined on the basis of this meta-analysis because glutamine was not part of the immunonutrition regimen in any included study. Furthermore, on subgroup analysis based on timing of initiation, significant beneficial effects of immunonutrition on postoperative total complications, infectious complications, and length of hospital stay were noted only in patients receiving postoperative administration. There was no significant difference in postoperative mortality between individual groups. The results act in cooperation with the enhanced recovery after surgery (ERAS) guidelines for perioperative care in patients undergoing hepatectomy [28].

It has been recommended that preoperative supplementation of immunonutrition for 5–7 days is required to improve the prognosis in major abdominal surgery [29, 30]. However, this meta-analysis did not reveal significant benefit from preoperative or perioperative immunonutrition. One reason for this may be attributed to a relatively small sample size of eligible RCTs, which masks the actual benefits of immunonutrition support in patients preoperatively or perioperatively. For this reason we cannot identify the optimal timing of the immunonutrition from this meta-analysis. Many included studies most often provided immunonutrition postoperatively, which may be partly due to high compliance after surgery for hospitalized patients.

GRADEpro analysis was performed to grade the quality of evidence and revealed an acceptable overall methodological quality of this meta-analysis (Supplementary Table 2). The strength of evidence for the postoperative total complications and infectious complications in patients receiving immunonutrition, and the evidence quality of postoperative total complications, infectious complications, and length of hospital stay in patients receiving ω-3 FAs were moderate, which concurred with the statistically significant benefit in the immunonutrition group. We downgraded the quality of evidence for infectious complications in patients receiving IMPACT because of the low sample size from the included studies.

Several confounding factors were apparent in this meta-analysis. First, the sample sizes in several studies were relatively small [18, 19], which may weaken the power of our conclusion. Second, categories of diseases and administration routes differed among studies, which may cause heterogeneity and confuse the effects. Third, there was no significant difference between the immunonutrition group and control group regarding body mass index (BMI) or weight in recorded studies. However, two of the RCTs [14, 16] did not report BMI or weight, raising a potential methodological issue because immunonutrition was found to be more effective in malnourished patients [31]. Fourth, differences in the definition criteria of outcome variables among the RCTs may result in imprecision. For example, two of the studies [13, 19] evaluated patients 30 days after surgery, whereas three studies [14, 15, 17] evaluated outcomes within 6 months. These differences in endpoints may have affected the outcomes of our meta-analysis. Therefore, a priori it was decided that studies were heterogeneous, and the random-effects model was applied.

In conclusion, our meta-analysis reveals that perioperative administration of immunonutrition in patients undergoing hepatectomy may reduce the postoperative total complications, infectious complications, and length of hospital stay. This improvement in the postoperative clinical outcome is of more benefit when ω-3 FA-enriched supplementation is provided. The GRADEpro approach has been used to assess the quality of evidence of this meta-analysis. However, methodological differences do exist among some studies and the number of patients included in present meta-analysis is relatively small. Additional RCTs with better methodological quality and larger sample size are needed before we can draw more robust conclusions.

## MATERIALS AND METHODS

This systematic review and meta-analysis of RCTs followed the guidelines of the Cochrane Handbook for Systematic Reviews of Intervention [32] and Preferred Reporting Items for Systematic Review and Meta-analysis (PRISMA) [33]. The prospective protocol, including the method and eligibility criteria, was registered in PROSPERO with the registration number CRD42017054166, and the quality of the study was evaluated using the PRISMA 2009 checklist (Supplementary Table 3).

### Literature search and selection

PubMed, Embase, the Cochrane Central Register of Controlled Trials (CENTRAL), and the Web of Science were searched for any RCT published through September 2016 that investigated the effects of perioperative immunonutrition support in patients undergoing hepatectomy. The search strategy for PubMed was based on the combination medical subject heading (MeSH) and free text: (“liver resection” [tiab] OR “hepatic resection” [tiab] OR “hepatectomy” [tiab]) AND (immunonutrition [tiab] OR “immune-enhancing diet” [tiab] OR “immune nutrients” [tiab] OR “fatty acids, omega-3” [mesh] OR“acids, omega-3 fatty” [tiab] OR “fatty acids, omega 3” [tiab] OR “omega-3 fatty acids” [tiab] OR “omega 3 fatty acids” [tiab] OR “n-3 PUFA” [tiab] OR “n-3 fatty acids” [tiab] OR “n-3 fatty acid” [tiab] OR “fatty acids, n-3” [tiab] OR “n 3 fatty acids” [tiab] OR “n-3 polyunsaturated fatty acid” [tiab] OR “n 3 polyunsaturated fatty acid” [tiab] OR “fish oil” [tiab] OR “arginine” [tiab] OR “glutamine” [tiab] OR “nucleotides” [tiab] OR “RNA” [tiab]). This search was supplemented by a manual search and by reviewing the reference lists of selected papers to avoid missing any articles. Only studies on humans and in English and Chinese were considered for inclusion. Two co-authors independently identified the studies for inclusion by reviewing their titles, abstracts, and full texts. Reviewers were not blinded to authors, institutions, or journals.

### Inclusion and exclusion criteria

The inclusion criteria were based on population, intervention, comparison, outcome, and study design (PICOS). Population (P): trials that included only patients who underwent hepatectomy. Intervention (I) and Comparison (C): trials comparing outcomes in patients with and without preoperative, perioperative, or postoperative immunonutrition supplementation that included at least one from ω-3 FA, arginine, glutamine, and nucleotides. Outcomes of interest (O): trials that assessed postoperative complications, length of hospital stay, and mortality. Design of study (S): RCT.

Articles were excluded if they were unoriginal studies, such as reviews, editorials, expert opinions, and articles without original data. Also excluded were abstracts, letters, case reports, and conference papers. Articles were also excluded if requisite information was lacking or original data could not be obtained from the authors, as well as those not fulfilling the inclusion criteria.

### Data extraction

Two independent co-authors collected data of interest from included studies, using a predesigned standard data extraction form (Supplementary Table 4). Any differences between the two authors were resolved by consensus. Collected data included the country, timing of intervention, duration of intervention, type and composition of study, control enteral diet, route of nutrition, analysis type, dropouts, sample size, age of patients, sex of patients, baseline BMI, duration of surgery, operative blood loss, Pringle time, and clinical diagnosis before surgery.

Outcomes of interest recorded included postoperative total complications, postoperative infectious complications, length of hospital stay, and postoperative mortality. Infectious complications included pulmonary or lower respiratory tract-related, surgical site-related, abdomen-specific, intravenous catheter-associated, and urinary tract-related infection as described in the studies.

### Quality assessment

Risk of bias of eligible articles was evaluated by two investigators according to the Cochrane Collaboration’s tool for assessing the risk of bias [32]. Bias was assessed by evaluating seven items: method of randomized sequence generation, method of concealing allocation, blinding of participants and personnel, blinding of outcome assessments, incomplete outcome data, selective reporting, and other potential items. Risk of bias was ranked as high, low, or unclear (Supplementary Table 5).

The quality of evidence for the outcome was evaluated by Grades of Recommendation, Assessment, Development, and Evaluation (GRADE) using GRADEpro software, version 3.6 (Evidence Prime, Hamilton, ON, Canada, 2015) [34]. These criteria were based on study design, risk of bias, inconsistency, indirectness, precision, publication bias, and other considerations. The quality of evidence was classified as high, moderate, low, or very low.

### Statistical analysis

All analyses were performed using RevMan 5.3.5 software (Cochrane Collaboration, Copenhagen, Denmark, 2013). For quantitative variables, the pooled effect was calculated as mean difference or SMD, along with the corresponding 95% CI. The outcomes for qualitative variables were analyzed to obtain a pooled RR with 95% CI. Study heterogeneity was defined using chi-squared (χ^2^) *p* value of < 0.10 or *I*^2^ > 50%. A pooled effect size was calculated using a random effects model with the inverse variance method, regardless of the presence or absence of heterogeneity. In all analyses, *p* < 0.05 was defined as statistically significant. The funnel plot was not produced to test the publication bias in this article due to the limited number (< 10) of studies included in each analysis [35].

## SUPPLEMENTARY MATERIALS TABLES









## Abbreviations

ASPEN: American Society for Parenteral and Enteral Nutrition
BMI: baseline body mass index
CI: confidence interval
ESPEN: European Society for Clinical Nutrition and Metabolism
GI: gastrointestinal
LoS: length of stay
MD: mean difference
RCT: randomized controlled trial
RR: risk rate
SD: standard deviation
SMD: standardized mean difference
