# Supplementary material for: The benefit of immunonutrition in patients undergoing hepatectomy: a systematic review and meta-analysis
Source: Oncotarget. 2017 Aug 8;8(49):86843–52. doi: 10.18632/oncotarget.20045 (PMC5689729; doi:10.18632/oncotarget.20045)
Supplement: Supplementary file 1 [file oncotarget-08-86843-s001.pdf]

The benefit of immunonutrition in patients undergoing hepatectomy: a systematic review and meta-analysis

SUPPLEMENTARY MATERIALS

Supplementary Table 1: Summary of the RCT studies included in this meta-analysis[1, 2]. See Supplementary_Table_1

Supplementary Table 2: Quality assessment based on component of perioperative immunonutrition support in patients undergoing hepatectomy[1]. See Supplementary_Table_2

Supplementary Table 3: PRISMA 2009 checklist. See Supplementary_Table_3

Supplementary Table 4: Basic characteristics of included into study

Study Information	Author		Year	Country	
Participants	Diagnosis				
	Age		Immunonutrition group	Control group	
	Sex		Immunonutrition group	Control group	
			Male: Female	Male: Female	
	Inclusion criteria				
	Exclusion criteria				
Interventions	Immunonutrition group	Content			
	Control group	Content			
Outcomes					
Notes					
Drop-Outs	Drop out due to		Immunonutrition group	Control group	
	the numbers of patients in the early stage				
	the numbers of patients in the late stage				
Continuous data					
Outcomes	Name of outcome		Data extraction		
			Mean	SD	N
	Immunonutrition group				
	Control group				
Binary data					
Outcomes	Name of outcome		Data extraction		
			Event number	Total number	
	Immunonutrition group				
	Control group				

Supplementary Table 5: Assessing of risk of bias tool

Item	Description	Risk of Bias
**Sequence Generation**	Describe the method used to generate the allocation sequence in sufficient detail to allow an assessment of whether it should produce comparable groups.	Was the allocation sequence adequately generated?
	Comment:	Unclear/High risk/Low risk
**Allocation Concealment**	Describe the method used to conceal the allocation sequence in sufficient detail to determine whether intervention allocations could have been foreseen.	Was the allocation adequately concealed?
	Comment:	Unclear/High risk/Low risk
**Blinding of Participants and Personnel**	Describe all measures used, if any, to blind study participants and personnel from knowledge of which intervention a participant received. Provide any information relating to whether the intended blinding was effective.	Was knowledge of the allocated intervention adequately prevented during the study?
	Comment:	Unclear/High risk/Low risk
**Blinding of Outcome Assessors**	Describe all measures used, if any, to blind outcome assessors from knowledge of which intervention a participant received. Provide any information relating to whether the intended blinding was effective.	Was knowledge of the allocated intervention adequately prevented during the study?
	Comment:	Unclear/High risk/Low risk
**Incomplete Outcome Data**	Describe the completeness of outcome data for each main outcome, including attrition and exclusions from the analysis. State whether attrition and exclusions were reported, the numbers in each intervention group (compared with total randomized participants), reasons for attrition/exclusions where reported, and any reinclusions in analyses performed by the review authors.	Were incomplete outcome data adequately addressed?
	Comment:	Unclear/High risk/Low risk
**Selective Outcome Reporting**	State how the possibility of selective outcome reporting was examined by the review authors and what was found.	Are reports of the study free of suggestion of selective outcome reporting?
	Comment:	Unclear/High risk/Low risk
**Other Bias**	State any important concerns about bias not addressed in the other domains in the tool. If particular questions/ entries were re-specified in the review protocol, responses should be provided for each question/entry	Was the study apparently free of other problems that could put it at high risk of bias?
	Comment:	Unclear/High risk/Low risk
