# Supplementary material for: The benefit of immunonutrition in patients undergoing hepatectomy: a systematic review and meta-analysis
Source: Oncotarget. 2017 Aug 8;8(49):86843–52. doi: 10.18632/oncotarget.20045 (PMC5689729; doi:10.18632/oncotarget.20045)
Supplement: Supplementary file 2 [file oncotarget-08-86843-s002.docx]

**Supplementary Table 1: Summary of the RCT studies included in this meta-analysis**^[[1]](#footnote-1)^1^,2^

| Study (ref) | Country | Timing of Intervention | Duration of Intervention, days | Type of Immunonutrition | Route of Nutrition |
| --- | --- | --- | --- | --- | --- |
| Gong et al., 2016[12] | China | postoperation | 5 | Omega-3 fatty acids (Omegaven, Sino-Swed) | PN/CVC |
| Zhang et al., 2016[13] | China | postoperation | 5 | Omega-3 fatty acids (Omegaven, Fresenius Kabi) | PN/CVC |
| Cockbain et al., 2014[14] | UK | preoperation | 12-65 | Omega-3 fatty acids eicosapentaenoic acid (ALFA, SLA) | EN/Oral |
| Wu et al., 2012[15] | China | postoperation | 5-7 | Omega-3 fatty acids (Omegaven, Fresenius Kabi) | PN/CVC |
| Wang et al., 2011[16] | China | postoperation | 6 | Omega-3 fatty acids (Omegaven, Sino-swed) | PN |
| Lai et al., 2012[17] | China | postoperation | 5 | Omega-3 fatty acids (Omegaven, Sino-Swed) | PN |
| Mikagi et al., 2011[18] | Japan | preoperation | 5 | Omega-3 fatty acids, arginine, nucleotides (IMPACT, Ajinomoto) | EN/Oral |
| Seguin et al., 2016[19] | France | perioperation | 10 | Omega-3 fatty acids, arginine, RNA (IMPACT, Novartis) | EN/Oral & nasogastric tube |

**Supplementary Table 1** (continued)

| Study (ref) | Intervention | | Analysis Type |
| --- | --- | --- | --- |
|  | IM | Con |  |
| Gong et al., 2016[12] | Omegaven 10% 100 mL/day rather than regular nutrition | Regular nutrition (fat emulsion, amino acids (17AA) and glucose (11%) injection (Kabiven^TM^ PI, Sino-Swed) 1920 mL/day | PP/ITT |
| Zhang et al., 2016[13] | Glucose, lipid emulsion (20% Structolipid and 10% n-3 fatty acid), amino acids, vitamins and electrolytes were compounded in an “All-In-One” manner | Glucose, lipid emulsion (20% Structolipid), amino acids, vitamins and electrolytes were compounded in an “All-In-One” manner | PP |
| Cockbain et al., 2014[14] | 1 g Omega-3 fatty acid eicosapentaenoic acid twice daily with food | 1 g mixed capric and caprylic acid medium-chain triglycerides twice-daily with food (Sasol) | ITT |
| Wu et al., 2012[15] | TPN (amino acids, glucose, a soybean oil emulsion with omegat-3 fatty acid supplementation) | TPN (amino acids, glucose, a soybean oil emulsion without omegat-3 fatty acid supplementation) | PP/ITT |
| Wang et al., 2011[16] | Amino acids, glucose, medium and long-chain lipid emulsion combined with omega-3 fatty acid | Amino acids, glucose, medium and long-chain lipid emulsion (Lipovenoes, Sino-swed) | PP/ITT |
| Lai et al., 2012[17] | 20% fish oil and 80% medium long lipid emulsion | 100% medium-long lipid emulsion | PP |
| Mikagi et al., 2011[18] | 750 ml/day IMPACT and half meals | Conventional hospital meals | PP |
| Seguin et al., 2016[19] | 900 ml/day IMPACT and regular food | Medium-chain triglyceride, protein(Novartis Nutrition, Bern, Switzerland) and regular food | PP |

**Supplementary Table 1** (continued)

| Study (ref) | Dropouts, *n* | | Patients, *n* | | Mean Age, year | | Sex, M:F | | BMI | | Duration of Surgery, min | |
| --- | --- | --- | --- | --- | --- | --- | --- | --- | --- | --- | --- | --- |
|  | IM | Con | IM | Con | IM | Con | IM | Con | IM | Con | IM | Con |
| Gong et al., 2016[12] | 0 | 0 | 59 | 60 | 51.37±11.75 | 49.63±11.21 | 44:15 | 46:14 | R^3^ | R^3^ | 267.9 ±112.0 | 289.4±147.5 |
| Zhang et al., 2016[13] | 3 | 5 | 160 | 160 | 47.59±11.69 | 49.62±11.85 | 115:42 | 112:43 | 63.14±8.51^4^ | 63.52±10.39^4^ | 271.92±112.07 | 282.04±89.28 |
| Cockbain et al., 2014[14] | 7 | 10 | 43 | 45 | 68 (44-82)^5^ | 71 (35-87)^5^ | 26:17 | 35:10 | - | - | - | - |
| Wu et al., 2012[15] | 0 | 0 | 31 | 32 | 52.00±7.90 | 52.16±8.31 | 25:6 | 25:7 | 22.47±1.80 | 22.64±1.63 | 214.2±58.8 | 218.4±55.8 |
| Wang et al., 2011[16] | 0 | 0 | 41 | 41 | 51.93 ±10.18 | 55.83±8.11 | 26:15 | 29:12 | - | - | - | - |
| Lai et al., 2012[17] | 8 | 6 | 40 | 40 | 50.3±10.2 | 53.0±15.6 | 34:6 | 32:8 | 20.8±2.60 | 21.3±2.60 | 164±39 | 171±42 |
| Mikagi et al., 2011[18] | 12 | 3 | 25 | 16 | 67.5±11.3 | 61.5±10.2 | 10:3 | 8:5 | 23.6±3.80 | 21.5±4.40 | 376.5±74.9 | 424.4±111.8 |
| Seguin et al., 2016[19] | 5 | 5 | 18 | 17 | 65±8 | 68±6 | 17:1 | 14:3 | 26.9±4.50 | 28.0±4.60 | - | - |

**Supplementary Table 1** (continued)

| Study (ref) | Operative Blood Loss, ml | | Pringle Time, min | | Clinical Diagnosis Before Surgery |
| --- | --- | --- | --- | --- | --- |
|  | IM | Con | IM | Con |  |
| Gong et al., 2016[12] | 423.3±395.8 | 451.4±243.3 | 18.6±12.0 | 16.7±9.2 | Primary liver cancer, metastatic liver cancer and benign liver diseases |
| Zhang et al., 2016[13] | 2.74±0.42^6^ | 2.73±0.42^6^ | 1.14±0.24^6^ | 1.20±0.22^6^ | HCC, intrahepatic cholangiocellular carcinoma and colorectal cancer liver metastases |
| Cockbain et al., 2014[14] | - | - | - | - | Colorectal cancer liver metastases |
| Wu et al., 2012[15] | 344±146 | 341±137 | 11.52±4.86 | 11.56±4.09 | HCC |
| Wang et al., 2011[16] | - | - | - | - | - |
| Lai et al., 2012[17] | 293±157 | 312±162 | 18.2±10.7 | 17.1±6.7 | HCC |
| Mikagi et al., 2011[18] | 823.5±667.8 | 723.3±490.4 | 20.1±20.6 | 14.9±15.5 | HCC, cholangiocellular carcinoma, metastatic liver cancer and benign liver diseases |
| Seguin et al., 2016[19] | - | - | - | - | Liver cancer with cirrhosis |

1. 1 Con, control; CVC, central venous catheter; EN, enteral nutrition; HCC, hepatocellular carcinoma; IM, immunonutrition; ITT, intention-to-treat analysis; n, sample size; PN, parenteral nutrition; PP, per-protocol analysis; TPN, total parenteral nutrition; ref, reference.

   ^2^ Results are expressed as means ± SD.

   ^3^ Report that there is no difference between two groups from stratified analysis.

   ^4^Results are expressed as weight (kg).

   ^5^Results are expressed as median (range).

   ^6^Results are expressed as Log format. [↑](#footnote-ref-1)
