# Supplementary material for: The benefit of immunonutrition in patients undergoing hepatectomy: a systematic review and meta-analysis
Source: Oncotarget. 2017 Aug 8;8(49):86843–52. doi: 10.18632/oncotarget.20045 (PMC5689729; doi:10.18632/oncotarget.20045)
Supplement: Supplementary file 3 [file oncotarget-08-86843-s003.docx]

**Supplementary Table 2: Quality** **assessment based on component of perioperative immunonutrition support in patients undergoing hepatectomy**^[[1]](#footnote-1)^1

| **Quality assessment** | | | | | | | | **Summary of findings** | | | | |
| --- | --- | --- | --- | --- | --- | --- | --- | --- | --- | --- | --- | --- |
| **Outcomes** | **Design** | **Limitations** | **Inconsistency** | | **Indirectness** | **Imprecision** | **Other considerations** | **No of patients** | | **Effect** | | **Quality** |
|  |  |  |  |  |  |  |  | **Immunonutrition** | **Control** | **RR (95% CI)** | **Absolute** |  |
| **Postoperative total complications** | | | | | | | | | | | | |
| ω-3 FAs | RCT | Serious^[[2]](#footnote-2)^2 | No serious | | No serious | No serious | None | 66/259 (25.5%) | 111/260 (42.7%) | RR 0.6 (0.46 to 0.76) | 171 fewer per 1000 (from 102 fewer to 231 fewer) | ⊕⊕⊕O MODERATE |
|  |  |  |  |  |  |  |  |  | 43.3% |  | 173 fewer per 1000 (from 104 fewer to 234 fewer) |  |
| IMPACT | RCT | Serious^2^ | No serious | | No serious | Serious^3^ | Serious^4^ | 1/25 (4%) | 3/16 (18.8%) | RR 0.21 (0.02 to 1.88) | 148 fewer per 1000 (from 184 fewer to 165 fewer) | ⊕OOO Very LOW |
|  |  |  |  |  |  |  |  |  | 18.8% |  | 149 fewer per 1000 (from 184 fewer to 165 fewer) |  |
| Immunonutrition | RCT | Serious^2^ | No serious | | No serious | No serious | None | 67/284 (23.6%) | 114/276 (41.3%) | RR 0.59 (0.46 to 0.75) | 169 fewer per 1000 (from 103 fewer to 223fewer) | ⊕⊕⊕O MODERATE |
|  |  |  |  |  |  |  |  |  | 31% |  | 127 fewer per 1000 (from 78 fewer to 167fewer) |  |
| **Postoperative infectious complications** | | | | | | | | | | | | |
| ω-3 FAs | RCT | Serious^2^ | | No serious | No serious | No serious | None | 30/290 (10.3%) | 62/292 (21.2%) | RR 0.49 (0.33 to 0.73) | 108 fewer per 1000 (from 57 fewer to 142 fewer) | ⊕⊕⊕O MODERATE |
|  |  |  |  |  |  |  |  |  | 22.7% |  | 116 fewer per 1000 (from 61 fewer to 152 fewer) |  |
| IMPACT | RCT | Serious^2^ | | No serious | No serious | Serious^3^ | None | 2/43 (4.7%) | 8/33  (24.2%) | RR 0.26 (0.07 to 0.95) | 179 fewer per 1000 (from 12 fewer to 225 fewer) | ⊕⊕OO LOW |
|  |  |  |  |  |  |  |  |  | 23.7% |  | 175 fewer per 1000 (from 12 fewer to 220 fewer) |  |
| Immunonutrition | RCT | Serious^2^ | | No serious | No serious | No serious | None | 32/333 (9.6%) | 70/325 (21.5%) | RR 0.46 (0.32 to 0.68) | 116 fewer per 1000 (from 69 fewer to 146 fewer) | ⊕⊕⊕O MODERATE |
|  |  |  |  |  |  |  |  |  | 22.7% |  | 123 fewer per 1000 (from 73 fewer to 154 fewer) |  |
| **Length of hospital stay** | | | | | | | | | | | | |
| ω-3 FAs | RCT | Serious^2,5^ | | No serious | No serious | No serious | None | 331 | 333 |  | SMD 0.49 lower (0.81 lower to 0.16 lower) | ⊕⊕⊕O MODERATE |
| **Postoperative mortality** | | | | | | | | | | | | |
| ω-3 FAs | RCT | Serious^2,6^ | | No serious | No serious | No serious | None | 4/333 (1.2%) | 11/337 (3.3%) | RR 0.46 (0.16 to 1.31) | 18 fewer per 1000 (from 27 fewer to 10 more) | ⊕⊕⊕O MODERATE |
|  |  |  |  |  |  |  |  |  | 2.2% |  | 12 fewer per 1000 (from 18 fewer to 7 more) |  |

1. 1RCT, Randomized controlled trial. [↑](#footnote-ref-1)
2. 2Studies fulfilling less than 3 elements for risk of bias are considered to have limitations in design.

   ^3^Low sample size.

   ^4^There is only 1 study for this outcome.

   ^5^ Discrepancy in the definition of length of hospital stay.

   ^6^Postoperative follow-up is diverse among studies. [↑](#footnote-ref-2)
